# Fatigue Damage Evaluation of Short Carbon Fiber Reinforced Plastics Based on Phase Information of Thermoelastic Temperature Change

**DOI:** 10.3390/s17122824

**Published:** 2017-12-06

**Authors:** Daiki Shiozawa, Takahide Sakagami, Yu Nakamura, Shinichi Nonaka, Kenichi Hamada

**Affiliations:** 1 Department of Mechanical Engineering, Kobe University, Kobe 657-8501, Japan; sakagami@mech.kobe-u.ac.jp (T.S.); 169t348t@stu.kobe-u.ac.jp (Y.N.); 2 DIC Corporation, Tokyo 103-8233, Japan; shinichi-nonaka@mb.dic.co.jp (S.N.); kenichi-hamada@mb.dic.co.jp (K.H.)

**Keywords:** nondestructive evaluation, thermoelastic stress analysis, phase analysis, infrared camera, short carbon fiber reinforced plastics

## Abstract

Carbon fiber-reinforced plastic (CFRP) is widely used for structural members of transportation vehicles such as automobile, aircraft, or spacecraft, utilizing its excellent specific strength and specific rigidity in contrast with the metal. Short carbon fiber composite materials are receiving a lot of attentions because of their excellent moldability and productivity, however they show complicated behaviors in fatigue fracture due to the random fibers orientation. In this study, thermoelastic stress analysis (TSA) using an infrared thermography was applied to evaluate fatigue damage in short carbon fiber composites. The distribution of the thermoelastic temperature change was measured during the fatigue test, as well as the phase difference between the thermoelastic temperature change and applied loading signal. Evolution of fatigue damage was detected from the distribution of thermoelastic temperature change according to the thermoelastic damage analysis (TDA) procedure. It was also found that fatigue damage evolution was more clearly detected than before by the newly developed thermoelastic phase damage analysis (TPDA) in which damaged area was emphasized in the differential phase delay images utilizing the property that carbon fiber shows opposite phase thermoelastic temperature change.

## 1. Introduction

Carbon fiber reinforced plastic (CFRP) has been widely employed for the primary structural members of transportation vehicles such as automobile, aircraft, or spacecraft, utilizing its excellent specific strength and specific rigidity in contrast with the metal. Short carbon fiber composite materials have been receiving a lot of attentions because of their excellent moldability and productivity. However, they show complicated behaviors in fatigue fracture due to their random fibers orientation. Therefore, effective nondestructive evaluation techniques are required for detecting and measuring various types of damages—such as fiber breakage, matrix cracking, and delamination—during fatigue fracture. Nondestructive evaluation techniques using infrared thermography, i.e., thermographic NDT has been effectively employed for the detection of delamination defects in fiber reinforced plastics. The thermographic NDT techniques based on thermal insulation effect by the delamination defect have been applied to the nondestructive testing of composite materials. The combination of active pulse heating and subsequent transient temperature measurement was effectively employed for sensitive detection of delamination damage in composites. Avdelidis et al. [1,2] reviewed NDT techniques using transient temperature distribution for CFRP as well as data processing techniques for pulsed thermography. Chatterjee et al. [3] compared the defect imaging performance among three transient thermography techniques, e.g., pulsed, lock-in, and frequency modulated thermography. Maldague et al. [4] developed pulse phase infrared thermography for improving the resolution of defect images and contributed to nondestructive evaluation of composite materials. As the transient temperature data analysis scheme, principal component analysis (PCA) has been investigated by several researchers to extract small changes in temperature trends [5,6,7]. 

In contrast with these thermal insulation techniques, damage evaluation techniques based on the thermoelastic stress analysis (TSA) using infrared thermography were examined for CFRP composites. TSA is a well-established experimental technique for the evaluation of the stress field in isotropic materials, and it has come into widespread use in the industry as an effective, experimental, full-field stress measurement technique [8,9,10,11,12]. Innovative research works on the TSA technique are found in structural integrity evaluations for steel structures related with fracture mechanics evaluations and/or fatigue damage analyses. As for steel structural members, evaluation schemes of the fracture mechanics parameters have been investigated, taking advantage of the TSA technique [13]. The fracture mechanics parameters such as stress intensity factor or J-integral have been directly determined from stress distribution around crack tips [14,15]. These techniques have been successfully applied to the fatigue life assessment for metallic materials. Tomlinson et al. [16] investigated fatigue crack propagation under mixed mode loading. Diaz et al. [17] applied their improved TSA technique for evaluating stress intensity factors in the fatigue test of weld specimen, demonstrating the feasibility of TSA for crack growth analysis influenced by crack closure or residual stress field.

Thermoelasticity for orthotropic materials have been studied by many researchers and the TSA technique has been employed as a powerful tool for evaluating impact or fatigue damage in composite materials and structures [18,19,20,21,22]. Paynter and Dutton [23] applied the TSA technique to wind turbine blade composite structure with successful results on damage evaluation using second harmonic signal correlation. Jones and Molent [24] showed the applicability of the TSA technique for in situ measurement during redesign, reinforcement or repair in aircraft structures. Emery and Dulieu-Barton [25] applied the TSA technique to fatigue damage evaluation in laminated glass fiber epoxy materials, in which TSA was employed as a powerful experimental tool for analyzing complicated fatigue damages such as fiber breakage, matrix cracking, and delamination damage in composites. Fruehmann and Dulieu-Barton [26] applied the TSA technique for the assessment of fatigue damage evolution in woven composite materials. Uenoya and Fujii [27] developed thermoelastic damage analysis (TDA) for the early damage detection in plain-woven CFRP. Yoshida et al. [28] applied TSA technique for the characterization of impact damages in cross-plied carbon fiber/thermoplastic composites.

The present authors [29] investigated the relationship between the fiber orientation angles and the phase delay of the thermoelastic temperature change from the applied loading signal. In this study, distributions of the thermoelastic temperature change and its phase delay were measured for short carbon fiber reinforced plastics during fatigue testing. The relationship between the phase difference and fiber orientation angle was investigated to develop a new thermoelasticity approach for fatigue damage identification for the short carbon fiber reinforced plastics. Fatigue damages were evaluated according to the conventional TDA procedure [27] as well as the newly developed phase-delay based damage characterizing technique “thermoelastic phase damage analysis (TPDA)”.

## 2. Thermoelastic Stress Analysis Using Infrared Thermography

Dynamic stress change cause a very small temperature change under adiabatic conditions in a solid. This phenomenon is known as the thermoelastic effect and is described by Lord Kelvin’s equation [30], which relates the temperature change (∆*T*_E_) to the sum of the changes in the principal stresses (∆*σ*) under cyclic variable loading as follows.

(1) $$\Delta T_{E}=-\frac{\alpha }{\rho C_{p}}T\Delta \sigma =-kT\Delta \sigma$$

Here, *α* is the coefficient of thermal expansion, *ρ* is the mass density, *C_p_* is the specific heat at constant pressure and *T* is the absolute temperature. The coefficient *k* is called thermoelastic constant. The sum of the changes in the principal stresses (∆*σ*) is obtained by measuring the temperature change (∆*T*_E_) using infrared thermography.

As thermoelastic temperature changes are very small and sometimes hidden by the thermal noise of the infrared camera, lock-in infrared thermography using reference signals synchronized with the stress changes is commonly employed to improve the accuracy of stress measurements. The TSA technique uses a lock-in algorithm with a reference-loading signal extracted from the load cell or strain gauge to improve the signal–noise ratio.

**Figure 1:**
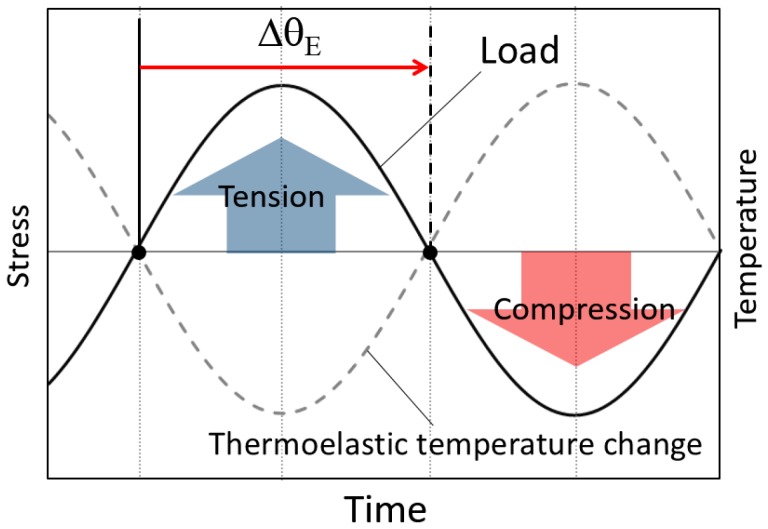
Definition of phase in thermoelastic temperature change.

When a tension and compression loading in sinusoidal waveform as shown in Figure 1 is applied to materials with positive value of thermoelastic constant *k*, the thermoelastic temperature change shows opposite phase waveform against the loading waveform. In this study, the value of the phase difference ∆*θ*_E_ was defined as the difference in phase between the thermoelastic temperature change and the loading signal as shown in the figure. In this case, phase difference ∆*θ*_E_ is 180 deg. In this study, thermoelastic temperature measurement was conducted for the short carbon fiber reinforced plastics, and the thermoelastic temperature change ∆*T*_E_ and the phase difference ∆*θ*_E_ was obtained from experimental data.

## 3. Experimental Setup

**Figure 2:**
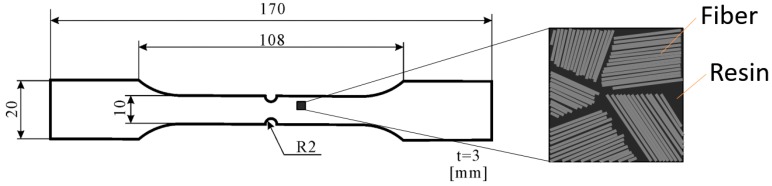
Configurations of employed short carbon fiber reinforced plastic specimen.

Configurations of the CFRP specimen employed in this study are shown in Figure 2. The specimens were cut from laminated short fiber CFRP sheet with vinyl ester resin and 25.4 mm long carbon fiber bundles. Each bundle was composed of 12,000 short carbon fibers. The mass content (wt %) of resin and fiber was 67 and 33, respectively. The specimen has circular notches with a radius of 2 mm.

Cyclic-axis sinusoidal waveform loading with a frequency *f* of 7 Hz and a stress ratio *R* = 0.1 was applied to the specimen by an electrohydraulic fatigue testing machine. Microscopic visible images on the specimen surface and side surface were taken by optical microscope. The temperature change on the specimen surface was measured by infrared thermography with an MCT array detector (CEDIP Inc. (FLIR Systems Inc.), Croissy Beaubourg, Fracnce (Wilsonville, OR, USA), Titanium530L). The specifications and setting of this infrared camera are listed in Table 1. The thermoelastic temperature change ∆*T*_E_ and the phase difference ∆*θ*_E_ were obtained from experimental data as shown in the forgoing paragraph.

## 4. Experimental Results

### 4.1. Effect of Fiber Orientation on Thermoelastic Temperature Change

**Figure 3:**
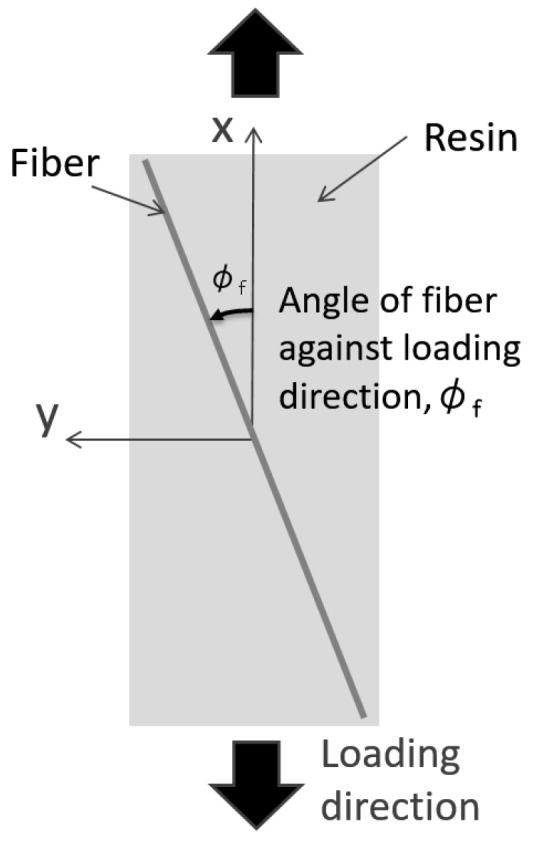
Definition of fiber orientation angle.

Before conducting fatigue test, the effect of the fiber orientation angle on the thermoelastic temperature change was experimentally investigated using a CFRP specimen without notches. Measurement of the thermoelastic temperature change was conducted under the applied maximum stress *σ*_max_ = 100 MPa. The fiber orientation angle *φ*_f_ is defined as shown in Figure 3; thus *φ*_f_ is equal to 0 degrees when a fiber bundle is oriented in parallel with the loading axis. The distribution of the fiber orientation angle *φ*_f_ was measured after the thermoelastic stress measurement as described below: (1) surface thin resin layer was removed by polishing for exposing carbon fiber bundles; (2) optical image of the surface carbon fiber bundles was taken by digital camera; (3) fiber orientation angle *φ*_f_ was determined by image processing program developed by Enomae [31].

**Figure 4:**
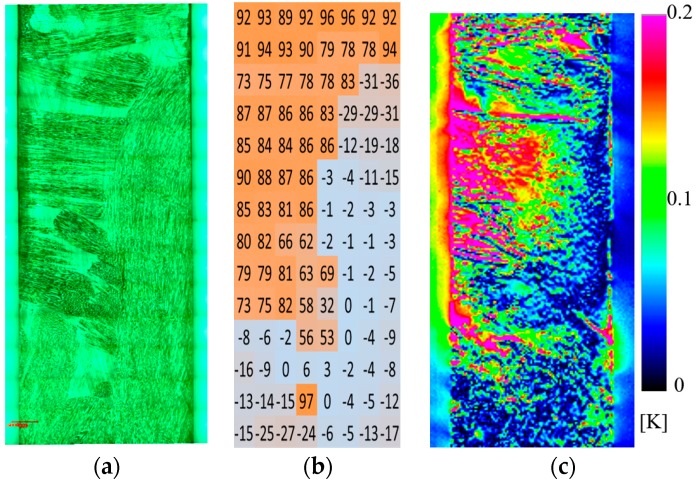
Fiber orientation angles and thermoelastic temperature change (*σ*_max_ = 100 MPa, *f* = 7 Hz): (**a**) optical image; (**b**) orientation angle; (**c**) thermoelastic temperature change.

**Figure 5:**
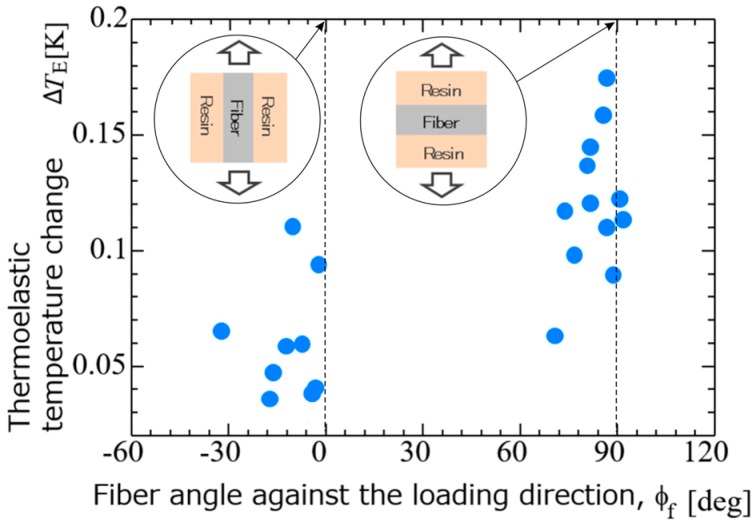
Relationship between thermoelastic temperature change ∆*T*_E_ and fiber orientation angle *φ*_f_.

**Figure 6:**
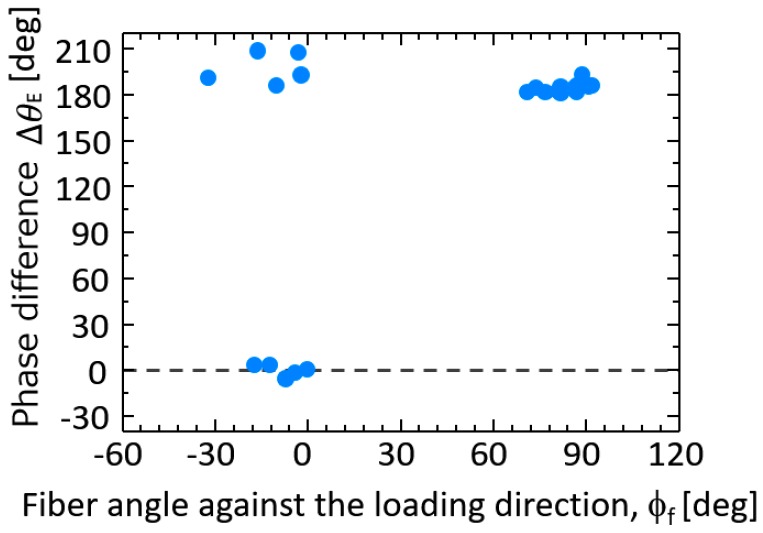
Relationship between phase difference ∆*θ*_E_ and fiber orientation angle *φ*_f_.: the phase difference ∆*θ*_E_ was measured for specimen in Figure 4 and Figure 5 under *σ*_max_ = 100 MPa and *f*=7 Hz.

Obtained optical image of the sample surface, distribution of fiber orientation angles, and thermoelastic temperature change ∆*T*_E_ obtained for CFRP specimen without notches are shown in Figure 4. The relationship between the thermoelastic temperature change and the fiber orientation angle *φ*_f_ is shown in Figure 5. It is found from these figures that ∆*T*_E_ measured in the area where *φ*_f_ is around 90° takes higher values compared with those measured in the area where *φ*_f_ is around 0°. Furthermore, the relationship between the fiber orientation angle *φ*_f_ and the phase difference ∆*θ*_E_ is shown in Figure 6. It can be seen from the figure that the phase difference ∆*θ*_E_ takes the value of 180° where the fiber orientation angles are around 90°. On the other hand, ∆*θ*_E_ takes 0° where *φ*_f_ is around 0°.

It was reported by Sugimoto and Ishikawa [32] that the coefficient of thermal expansion of carbon fiber in longitudinal direction has negative value, hence the thermoelastic constant *k* of unidirectional CFRP takes negative value. It is supposed that carbon fibers mainly shared the applied axial load in the region where the orientation angles of the carbon fiber bundles are 0° (parallel with the loading axis). Therefore, the thermoelastic temperature change showed a coordinate-phase waveform with that of applied loading.

### 4.2. Fatigue Damage Identification Based on Thermoelastic Temperature Change and Its Phase Delay Information

**Figure 7:**
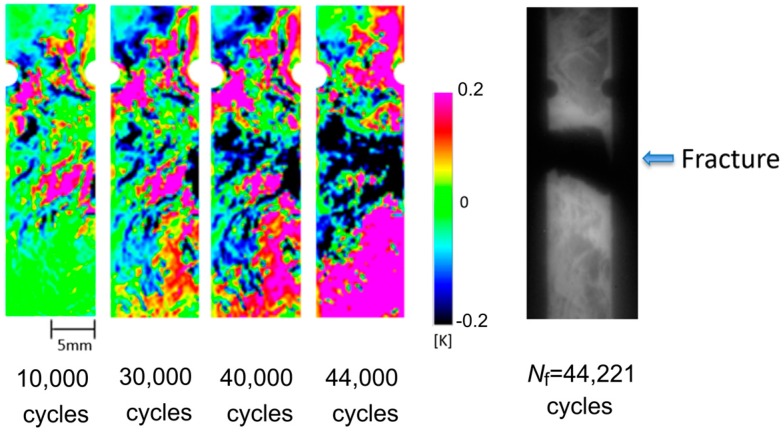
TDA images and infrared image obtained after specimen failure (*σ*_max_ = 180 MPa, *f* = 7 Hz).

Fatigue test was conducted for the short carbon fiber reinforced plastic specimen with circular notches under the applied maximum stress *σ*_max_ = 180 MPa. As described in the introduction, Uenoya et al. [25] developed the thermoelastic damage analysis (TDA) for the early damage detection in plain-woven long carbon fiber reinforced plastics. In this technique, a differential thermoelastic temperature distribution image was generated by subtracting an image of thermoelastic temperature change obtained at certain loading cycle from a reference initial image of thermoelastic temperature change. Fatigue damage evolution causes local stress change, and this is emphasized in the differential thermoelastic temperature distribution image. Therefore, the fatigue damage in CFRP sample can be detected in the differential thermoelastic temperature distribution image at the early stage in the fatigue life. In this study, the reference initial image of thermoelastic temperature change was set at 200 cycles. The obtained TDA images at 10,000; 30,000; 40,000; and 44,000 cycles are shown in Figure 7, with the infrared image obtained after failure of the CFRP specimen at 44,221 cycles. It is found from TDA images that the thermoelastic temperature change ∆*T*_E_ on the fracture part was decreasing with the increasing loading cycles. And the decreasing area of ∆*T*_E_ was expanded in the transverse direction of the specimen. This indicates that fatigue damage can be detected from the local stress change caused by the change in load sharing conditions due to the damage evolution.

**Figure 8:**
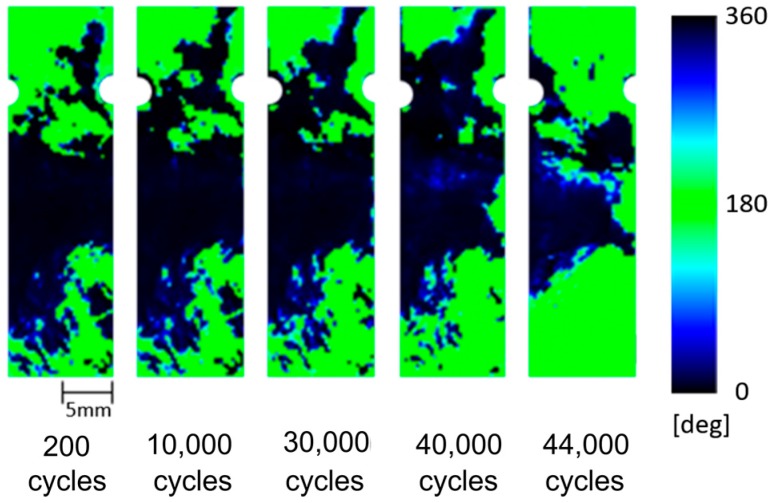
Distribution of phase delay measured at increasing loading cycles (*σ*_max_ = 180 MPa, *f* = 7 Hz).

**Figure 9:**
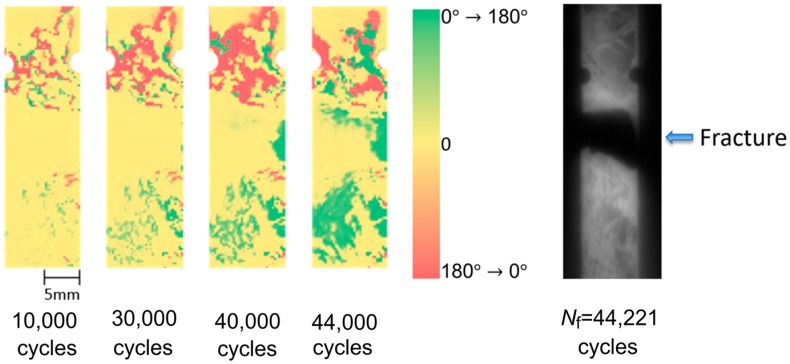
Results of thermoelastic phase damage analysis (TPDA) indicating early fatigue damage evolution (*σ*_max_ = 180 MPa, *f* = 7 Hz).

Distribution of the phase difference ∆*θ*_E_ obtained at 200; 10,000; 30,000; 40,000; and 44,000 cycles are shown in Figure 8. Change in phase values were found in the region where the structural fracture of CFRP sample was detected. The phase values were changed from 0 to 180°, and the phase change region was expanded with the increasing loading cycles. As described in the foregoing paragraph, ∆*θ*_E_ = 0° means that the thermoelastic effect of carbon fiber bundles is predominant, on the other hand ∆*θ*_E_ = 180° means that the thermoelastic effect of matrix resin is predominant. The change in phase values indicates the change of load sharing conditions between resin and carbon fiber bundles due to the fatigue damage evolution. We thought that high-sensitivity early fatigue damage detection technique can be developed by utilizing the change in phase difference ∆*θ*_E_; we propose a thermoelastic phase damage analysis (TPDA). In TPDA a differential phase delay distribution image is generated by subtracting a phase delay image obtained at certain loading cycle from a reference initial phase image. In this study the reference initial phase delay image was set at 200 cycles. The obtained TPDA images at 10,000; 30,000; 40,000; and 44,000 cycles are shown in Figure 9. It was found from TPDA images that the significant change of phase values from 0 to 180° (indicated green color in the figures) was found in the region where the structural fracture was detected.

**Figure 10:**
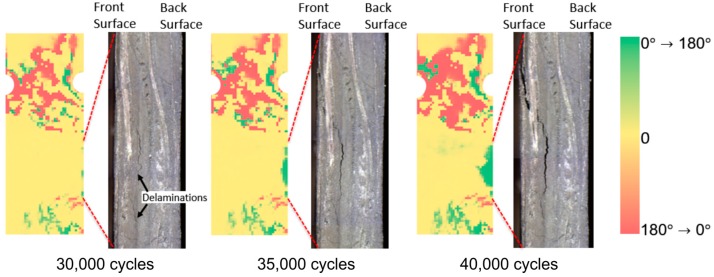
Microscopic visible images of specimen side surface indicating delamination damage evolution (*σ*_max_ = 180 MPa, *f* = 7 Hz).

In this experiment, visible images on the side surfaces of the CFRP specimen were taken by digital microscope. Microscopic visible images taken at 30,000; 35,000; and 40,000 loading cycles are shown in Figure 10 compared with TPDA results. The microscopic imaging area was indicated by red broken lines in the figures. It was found that delamination damage was initiated at 30,000 cycles and it grew and reached the front surface of the specimen (infrared observation side). TPDA images show the change in phase values was clearly detected in the region where the delamination fracture was initiated. The phase values were changed from 0 to 180°, and the phase change region was expanded with the increasing loading cycles. As described in the foregoing paragraph, ∆*θ*_E_ = 0° means that the thermoelastic effect of carbon fiber bundles is predominant, on the other hand ∆*θ*_E_ = 180° means that the thermoelastic effect of matrix resin is predominant. The change in phase values indicates the change of load sharing conditions between resin and carbon fiber bundles due to the evolution of delamination damage.

## 5. Conclusions

In this study, TSA using an infrared thermography was applied to the evaluation of fatigue damage in short carbon fiber composites. In addition to the conventional thermoelastic damage analysis, the phase difference between the thermoelastic temperature change and the applied loading signal was measured for short carbon fiber reinforced plastics. Obtained results are summarized as follows:

(1) It was found from the relationship between the thermoelastic temperature change ∆*T*_E_ and the fiber orientation angle *φ*_f_ that ∆*T*_E_ in the area where *φ*_f_ = 90° takes higher value compared with that in the area where *φ*_f_ = 0°.
(2) It was also found from the relationship between the fiber orientation angle *φ*_f_ and the phase difference ∆*θ*_E_ that ∆*θ*_E_ takes 180° in the area where *φ*_f_ = 90°. On the other hand, ∆*θ*_E_ takes 0° where *φ*_f_ = 0°. This is due to the negative thermoelastic constant of carbon fibers and the load sharing condition between resin and carbon fibers.
(3) Fatigue damage was evaluated according to the conventional TDA procedure as well as the newly developed phase-delay based damage characterizing technique “thermoelastic phase damage analysis (TPDA)”. It was found from TPDA images that the significant change of phase values from 0 to 180° (indicating the change in load sharing condition between resin and carbon fibers due to fatigue damage evolution) was found in the region where the structural fracture was detected.

## Figures and Tables

**Table 1:** Specifications and setting of employed infrared camera

Infrared Detector	MCT
Detectable wavelength	7.7–9.3 μm
Number of detectors	320 × 256
Temperature resolution (NETD)	25 mK
Framing rate	373 Hz
Time of data acquisition	10 s
